# Renewable Resources and a Recycled Polymer as Raw Materials: Mats from Electrospinning of Lignocellulosic Biomass and PET Solutions

**DOI:** 10.3390/polym10050538

**Published:** 2018-05-17

**Authors:** Rachel Passos de Oliveira Santos, Patrícia Fernanda Rossi, Luiz Antônio Ramos, Elisabete Frollini

**Affiliations:** Macromolecular Materials and Lignocellulosic Fibers Group, Center of Research on Science and Technology of BioResources, Institute of Chemistry of São Carlos, University of São Paulo, CP 780, 13560-970 São Carlos, SP, Brazil; rachelpassos@gmail.com (R.P.O.S.); patricia.rossi@usp.br (P.F.R.); ramos@iqsc.usp.br (L.A.R.)

**Keywords:** lignocellulosic sisal fibers, recycled PET, electrospinning, mechanical properties

## Abstract

Interest in the use of renewable raw materials in the preparation of materials has been growing uninterruptedly in recent decades. The aim of this strategy is to offer alternatives to the use of fossil fuel-based raw materials and to meet the demand for materials that are less detrimental to the environment after disposal. In this context, several studies have been carried out on the use of lignocellulosic biomass and its main components (cellulose, hemicelluloses, and lignin) as raw materials for polymeric materials. Lignocellulosic fibers have a high content of cellulose, but there has been a notable lack of investigations on application of the electrospinning technique for solutions prepared from raw lignocellulosic biomass, even though the presence of cellulose favors the alignment of the fiber chains during electrospinning. In this investigation, ultrathin (submicrometric) and nanoscale aligned fibers were successfully prepared via electrospinning (room temperature) of solutions prepared with different contents of lignocellulosic sisal fibers combined with recycled poly(ethylene terephthalate) (PET) using trifluoroacetic acid (TFA) as solvent. The “macro” fibers were deconstructed by the action of TFA, resulting in solutions containing their constituents, i.e., cellulose, hemicelluloses, and lignin, in addition to PET. The “macro” sisal fibers were reconstructed at the nanometer and submicrometric scale from these solutions. The SEM micrographs of the mats containing the components of sisal showed distinct fiber networks, likely due to differences in the solubility of these components in TFA and in their dielectric constants. The mechanical properties of the mats (dynamic mechanical analysis, DMA, and tensile properties) were evaluated with the samples positioned both in the direction (*dir*) of and in opposition (*op*) to the alignment of the nano and ultrathin fibers, which can be considered a novelty in the analysis of this type of material. DMA showed superior values of storage modulus (*E*’ at 30 °C) for the mats characterized in the preferential direction of fiber alignment. For example, for mats obtained from solutions prepared from a 0.4 ratio of sisal fibers/PET, Sisal/PET_0.40_*dir* presented a high *E*’ value of 765 MPa compared to Sisal/PET_0.40_*op* that presented an *E*’ value of 88.4 MPa. The fiber alignment did not influence the *T*_g_ values (from tan δ peak) of electrospun mats with the same compositions, as they presented similar values for this property. The tensile properties of the electrospun mats were significantly impacted by the alignment of the fibers: e.g., Sisal/PET_0.40_*dir* presented a high tensile strength value of 15.72 MPa, and Sisal/PET_0.40_*op* presented a value of approximately 2.5 MPa. An opposite trend was observed regarding the values of elongation at break for these materials. Other properties of the mats are also discussed; such as the index of fiber alignment, average porosity, and surface contact angle. To our knowledge, this is the first time that the influence of fiber alignment on the properties of electrospun mats based on untreated lignocellulosic biomass combined with a recycled polymer, such as PET, has been evaluated. The mats obtained in this study have potential for diversified applications, such as reinforcement for polymeric matrices in nanocomposites, membranes for filtration, and support for enzymes, wherein the fiber alignment, together with other evaluated properties, can impact their effectiveness in these applications.

## 1. Introduction

Renewable resources are viable, biodegradable, and low-energy consumption raw materials. They are a current subject of interest due to the benefits of increasingly replacing fossil-based raw materials in the preparation of polymeric materials [1,2,3,4,5,6,7,8].

Use of lignocellulosic fibers as reinforcing agents in polymeric composites is an example of the successful application of renewable resources [9,10,11,12,13,14]. In a previous study, composites based on recycled poly(ethylene terephthalate) (PET) reinforced with sisal fibers were prepared via a thermopressing process [9]. In this study, plasticizers derived from renewable raw materials were used to decrease the melting point of the recycled PET, which is sufficiently high to initiate thermal decomposition of the lignocellulosic fibers. Therefore, composites were successfully prepared from a recycled polymer that is widely available globally and from other components derived from renewable sources, i.e., sisal fibers and plasticizers [9]. Given our continued interest in recycled PET and lignocellulosic sisal fibers, the study progressed with the aim of continuously adding more value to these two raw materials, and this paper is focused on the electrospinning of solutions obtained from both of them.

The electrospinning technique has gained much attention because it is a versatile method that enables the production of submicro- and nanoscale fibers, including from biopolymer solutions [15,16,17,18]. However, there is a lack of investigations on the application of this technique to produce fibers based on raw lignocellulosic biomass [19,20], probably due to the shortage of solvents able to deconstruct the fibers to generate solutions of their components (cellulose, hemicelluloses, and lignin) that simultaneously have properties suitable for electrospinning. The linear structure of cellulose, the most abundant biopolymer in the world, favors the alignment of its chains during the electrospinning process, as opposed to hemicelluloses, which are heteropolysaccharides composed of branched chains. Lignin is a large-scale renewable source of aromatic functionalities, which gives it unique properties. However, the complex nonlinear structure of lignin prevents the electrospinning of lignin solutions [21].

Lignocellulosic sisal fibers have a high content of cellulose, which favors the electrospinning of hemicelluloses and lignin when the three components are together. Brazil is the world’s largest producer of sisal with a production in 2016 of 84.551 tons, and approximately 70% of the fibers produced were exported [21,22].

Rodrigues et al. [19] first reported the electrospinning of untreated lignocellulosic biomass using trifluoroacetic acid (TFA) as a solvent. The sisal fibers were deconstructed by dissolution and reconstructed as a homogeneous network of ultrathin and nanofibers.

PET is one of the major polymers recycled and reused for a wide range of applications [9]. This polymer, both recycled and new, has also been widely used as a raw material for the electrospinning process to produce ultrathin fibers [21,23,24,25]. These fibers can be used, e.g., in combination with graphitic carbon nitride as an easily recycled photocatalyst for the degradation of antibiotics under solar irradiation [26]

Santos et al. [20] pioneered the combination of lignocellulosic sisal fibers and recycled PET to produce non-aligned ultrathin fibers and nanofibers. Hybrid mats composed of non-aligned fibers were successfully prepared with different final properties than those presented by PET electrospun mats.

The electrospinning technique can also be successfully used to produce aligned fibers with a high degree of orientation [27,28,29]. Those fibers can present remarkable properties [30,31,32,33] and can be used in applications such as reinforcing agents in nanocomposites [33,34,35,36], as well as substituents of extracellular matrices (ECMs) in areas such as tissue engineering [37].

In the present study, mats composed of aligned fibers and based on lignocellulosic sisal fibers/recycled PET were produced, and their mechanical and surface properties were evaluated.

## 2. Materials and Methods

### 2.1. Materials

The recycled PET (MFI of 36.4 g·(10 min)^−1^ [9]) was a donation from Gruppo Mossi & Ghisolfi (M&G, São Paulo, Brazil).

The sisal fibers (chemical composition of 64.9% cellulose; 11.7% lignin; 25.4% hemicellulose; 7.1% moisture; 0.4% ash content; crystallinity index of 58% [9]) were acquired from Sisal Sul Indústria e Comércio LTDA (São Paulo, Brazil). Prior to their use, the lignocellulosic fibers were submitted to an extraction process using a mixture of ethanol/cyclohexane (experimental conditions: 1:1, *v*/*v*, 10 min). This pretreatment aimed to remove waxes (composed mostly of apolar molecules) and inorganic compounds. The extracted fibers were milled in a MARCONI MA048 (São Paulo, Brazil) vertical rotor mill (30 mesh stainless steel) and dried in a vacuum oven at 60 °C until they reached a constant weight. Trifluoroacetic acid (TFA) was purchased from Mallinckrodt Chemicals (Dublin, Ireland), and it was used as received.

### 2.2. Electrospinning Process

The electrospun solutions were prepared based on the study by Santos et al. [20]. Briefly, a reference solution of recycled PET (15 g·dL^−1^) and four solutions containing mixtures with different ratios of this polymer and sisal fibers were prepared, as summarized in Table 1. In all cases, the solutions were kept under vigorous magnetic stirring at room temperature until complete dissolution of the components (one-phase solution) was reached.

The concentration of sisal fibers was chosen based on a previous study on non-aligned electrospun mats [19], where it was found that 2 g·dL^−1^ corresponded to the threshold to avoid gelation after dissolution. The sisal fiber-based solutions presented high viscosity, which prevented determine the viscosities of the electrospun solutions using the falling-ball viscometer from GILMONT Instruments (Cole-Parmer scientific experts, Vernon Hills, IL, USA). The high volatility of the TFA represents a major drawback to the use of other methods to determine the viscosity of the solutions, such as the capillary viscometry.

**Figure 1:**
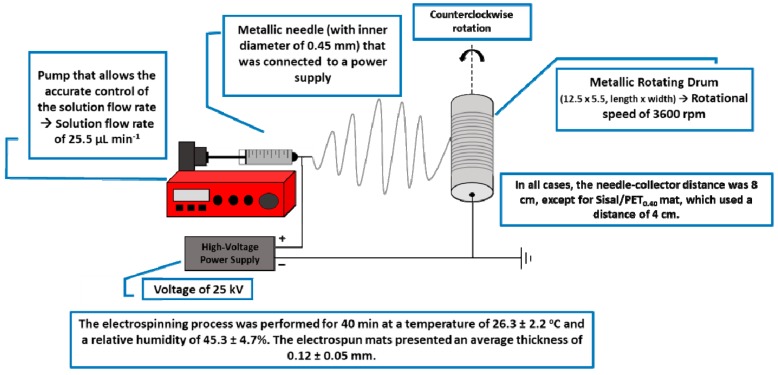
Experimental scheme with the electrospinning parameters adopted.

The electrospinning process was performed using an EC-DIG electrospinning apparatus from IME Technologies (Geldrop, The Netherlands) and a metallic rotator drum from Instor (Porto Alegre, RS, Brazil). All of the electrospinning conditions adopted, such as the needle-collector distance, voltage, solution flow rate, rotational speed of the collector and process time, were established after previous tests in which the viscosity of the solutions, lack of formation of beads, and diameter of the fibers were also taken into account. The one-phase solutions were placed into capillary tubes (ID: 1.00 mm and OD: 1.6 mm) and these tubes were connected to a mechanical pump (IME Technologies, Geldrop, The Netherlands). Figure 1 shows a scheme of the electrospinning parameters adopted in all experiments.

### 2.3. Characterization of the Materials

Scanning electron microscopy (SEM) was performed on a 440 Zeiss DSM 940 instrument (Oberkochen, Germany) using an accelerating voltage of 20 kV. Prior to this analysis, to improve the image acquisition, the samples were coated with an ultrathin gold layer using a sputter-coating system. ImageJ 1.45 image processing software (National Institutes of Health, Bethesda, MD, USA) was used to process and analyze the SEM images, including determination of the average fiber diameter, the average pore area and porosity, and the alignment index and average preferred orientation of the fibers.

The contact angle (25 °C) between droplets of deionized water and the surface of the mats were obtained from a CAM 200 goniometer (KSV Instruments Ltd., Helsinki, Finland) at Bernhard Gross Physics Institute of São Carlos, University of São Paulo, equipped with image analysis software (CAM 2008). Drops of water (with a volume of approximately 5 μL) were deposited on the surface of the mats (1 cm × 1 cm), and the contact angles (left and right) were calculated from the digitalized image. For this analysis, 500 measurements were individually collected at one second intervals, which allowed calculation of the resulting angle as a function of time. All measurements were carried out at least three times for each mat.

Dynamic mechanical analysis (DMA) was carried out on a thermal analyzer model Q800 from TA Instruments (New Castle, DE, USA) equipped with a tension clamp for films in multifrequency mode. The samples (6.3 mm in width and 5 mm in gauge length) were analyzed using the following parameters: amplitude of 4 μm, frequency of 1 Hz, static force of 0.25 N and heating rate of 3 °C·min^−1^ (from 0 to 200 °C). At least three specimens were tested from each sample group.

Tensile tests were performed at 25 °C using a TA Instruments model Q800 in tension mode. The samples (6.4 mm wide with a gauge length of 5 mm) were strained to 18 N or failure at a constant rate of 1 N·min^−1^. These tests were carried out with at least three specimens from each sample group.

The mechanical properties of the mats (DMA and tensile properties) were evaluated with the samples positioned both in the direction (*dir*) of and in opposition (*op*) to the alignment of the nano and ultrathin fibers to evaluate the influence of fiber alignment on these properties.

## 3. Results and Discussion

Mats composed of aligned fibers were produced via an electrospinning technique from solutions of a renewable resource—namely, sisal fibers—and a recycled polymer, i.e., recycled PET. These mats were characterized regarding their morphological and surface properties, as well as with respect to their mechanical properties, in order to evaluate the influence of fiber alignment on these properties.

**Figure 2:**
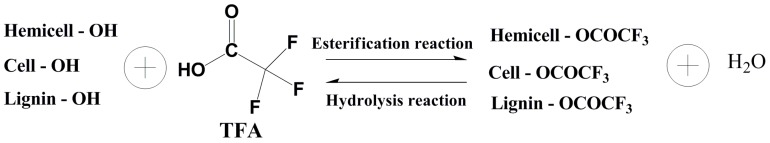
Reactions of esterification and hydrolysis of the lignocellulosic components.

TFA, the solvent used to dissolve the recycled polymer and to deconstruct the lignocellulosic fibers into their components, can esterify the hydroxyl groups present in the chemical structures of cellulose, hemicelluloses, and lignin, as shown in Figure 2. In a previous study, mats composed of sisal fiber solutions were analyzed via Fourier transform infrared spectroscopy (FTIR). The initial FTIR spectrum showed, in addition to the characteristic bands of a typical lignocellulosic material, a small shoulder at 1790 cm^−1^, which could be attributed to the trifluoroacetyl groups resulting from esterification [17], as shown in Figure 2.

However, exposure to air led to hydrolysis of the trifluoroacetyl groups resulting from esterification (hydrolysis reaction, Figure 2) [17], regenerating the constituents of the lignocellulosic biomass (Cell-OH, Hemicell-OH, Lignin-OH, Figure 2).

### 3.1. Scanning Electron Microscopy (SEM)

**Figure 3:**
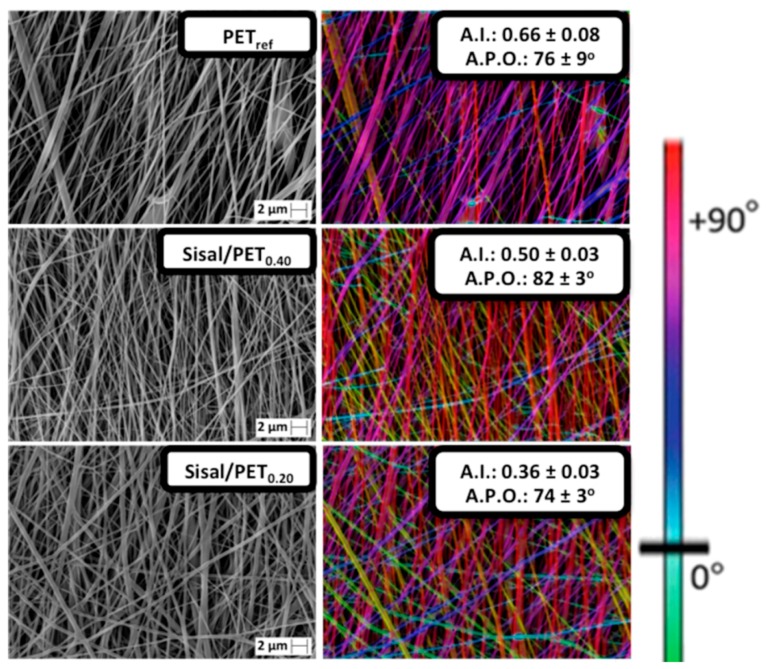

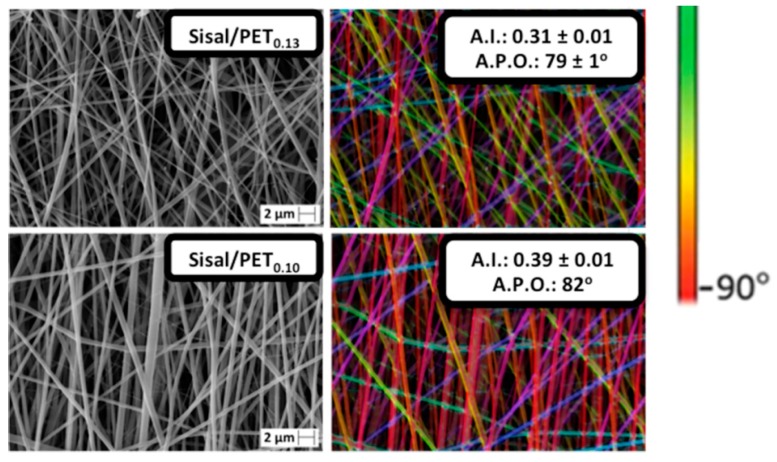
SEM micrographs of the electrospun mats and their respective color-coded images, alignment index (**AI**) and average preferred orientation (**APO**) of the fibers.

Figure 3 displays the SEM micrographs, respective color-coded images and data related to the alignment index (A.I.) and average preferred orientation (A.P.O.) of the electrospun mats PET_ref_, Sisal/PET_0.10_, Sisal/PET_0.13_, Sisal/PET_0.20_, and Sisal/PET_0.40_.

**Figure 4:**
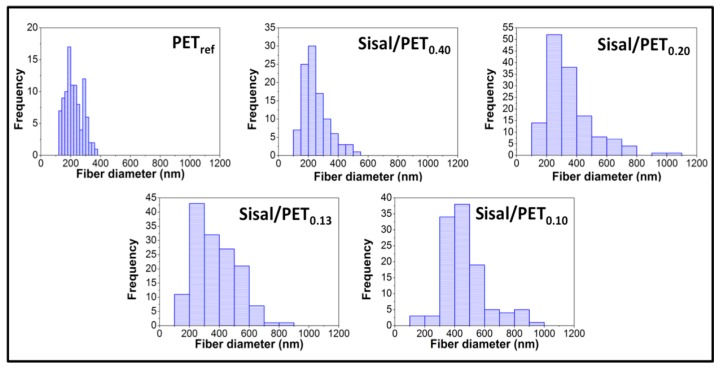
Histograms of fiber diameter frequency for the electrospun mats.

Figure 4 shows the histograms related to the fiber diameter frequency of the electrospun mats.

Few beads were observed in the Sisal/PET_0.13_ fiber network. The presence of beads along the fibers can be attributed to instability of the jet of polymer solution during the electrospinning process, as indicated by Yarin [38]. The formation of beads was also observed in the PET/sisal-based mats produced using the stationary fiber collector [20].

The SEM micrographs of the mats containing sisal in their compositions (mainly for Sisal/PET_0.10_, Figure 3) showed two distinct fiber networks. The fiber network with thicker fibers corresponded to fibers from recycled PET (as observed in the SEM micrograph of this polymer (Figure 3, PET_ref_.)), and the other network composed of thinner fibers probably corresponded to the components of the sisal fibers. The phenomenon of separate electrospinning of recycled PET and sisal fibers observed in the PET/sisal fiber-based mats (Figure 3) can be attributed to the differences in the solubility of these components in TFA and in their dielectric constants (parameters that can affect the electrospinning process of hybrid solutions). Additionally, PET and components of the sisal fibers may have partially electrospun together, generating hybrid fibers with an intermediate diameter between those mentioned above

**Figure 5:**
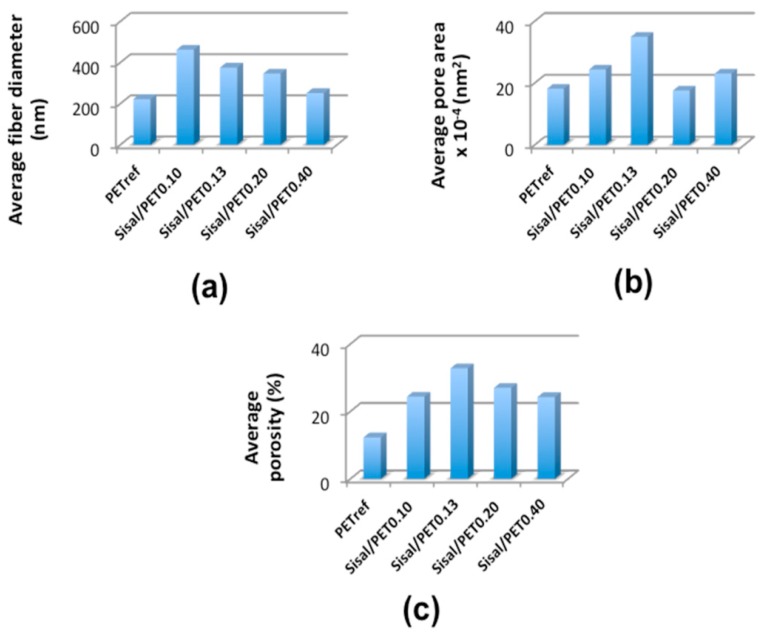
Average values of (**a**) fiber diameter (errors from ±59.10 (PET_ref_) to ±160.70 (Sisal/PET_0.20_)); (**b**) pore area (errors from ±1.00 (Sisal/PET_0.10_) to ±17.80 (Sisal/PET_0.40_)); and (**c**) porosity (errors from ±0.20 (PET_ref_) to ±7.10 (Sisal/PET_0.40_)) of the electrospun mats.

Regarding the histograms of fiber diameter frequency in the electrospun mats, it was observed that the materials containing sisal in their compositions presented a larger distribution of fiber diameters than PET_ref_, as shown in Figure 4. This was the result of the possibility that at least part of the components of the lignocellulosic sisal fibers were electrospun in combination with the PET chains (probably mostly lignin due to its aromatic structure and affinity for the aromatic structure of PET), in addition to having electrospun separately from the PET chains, as mentioned above. Thus, mats containing sisal in their compositions presented a network formed by thicker fibers (251.9 ± 87.5 nm (Sisal/PET_0.40_), 346.7 ± 160.7 nm (Sisal/PET_0.20_), 375.8 ± 143.2 nm (Sisal/PET_0.13_) and 462.9 ± 145.4 nm (Sisal/PET_0.10_) (Figure 5a)) and another one composed of fibers so thin that their diameters were below the threshold for determination via the ImageJ 1.45 image processing software. It should be highlighted that the electrospun raw lignocellulosic sisal fibers and sisal pulp fibers presented, respectively, a web of ultrathin fibers with diameters ranging from 120–510 nm, and nano/ultrathin fibers with diameters ranging from 65–200 nm, depending on the different values of solution flow rate adopted [17]. Further evidence of the large distribution of fiber diameters (Figure 4) presented by the sisal-based mats came from comparison between the standard deviations of the average diameter of PET_ref_ (±59.10 nm) and of the mats containing sisal fibers (±87.5, ±160.7, ±143.2, ±145.4 nm for Sisal/PET_0.40_, Sisal/PET_0.20_, Sisal/PET_0.13_ and Sisal/PET_0.10_, respectively).

The alignment index, A.I., ranged from 0.31 ± 0.01 (Sisal/PET_0.13_) to 0.66 ± 0.08 (PET_ref_), Figure 3. The differences observed between PET_ref_ and the Sisal/PET mats were probably due to the higher viscosity of the Sisal/PET solutions compared to PET_ref_, which led to an increase in the viscoelastic forces and hence to a decrease in the stretching and alignment of the fibers. Regarding the average preferred orientation (A.P.O.), there was no significant difference between any of the electrospun mats, as seen in Figure 3. Figure 5 presents the results of the average fiber diameter, average pore area, and average porosity of the electrospun mats.

The average pore areas ranged from 35.0 ± 4.2 × 10^4^ nm^2^ (Sisal/PET_0.13_) to 17.6 × 10^4^ nm^2^ (Sisal/PET_0.20_), which included PET_ref_ (18.2 ± 1.7 × 10^4^ nm^2^) (Figure 5b), showing that most of the Sisal/PET mats had higher average pore areas than PET_ref_. The same trend was observed when comparing the values of average porosity of the electrospun mats, which ranged from 12.3 ± 0.2% (PET_ref_) to 32.8 ± 6.9% (Sisal/PET_0.13_), as shown in Figure 5c. These results can be attributed to the network formed by the lignocellulosic components in the electrospun Sisal/PET-mats. The fibers present in this network presented smaller diameters, as mentioned above, which impacted both the average porosity and average pore area [39]. This high porosity combined with the high specific surface area and the low basic weight presented by this type of material, makes it suitable for use as a high-performance air filter, for example [40,41,42].

### 3.2. Contact Angle (CA) Measurements

Evaluation of the hydrophobic and hydrophilic characteristics of the electrospun mats is important for predicting the potential applications of these materials. For filtration purposes, for example, depending on the object to be intercepted, the ability to control the hydrophobic/hydrophilic characteristics of the mat can be very useful.

**Figure 6:**
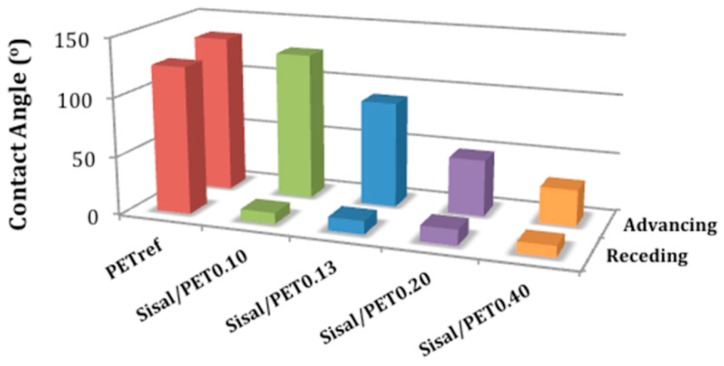
Advancing (errors from ±0.52 (PET_ref_) to ±16.31 (Sisal/PET_0.10_)) and receding (errors from ±2.21 (Sisal/PET_0.10_) to ±7.53 (Sisal/PET_0.13_)) contact angles between the water droplet and the surface of the electrospun mats.

The advancing and receding contact angles between the water droplets and the surfaces of the electrospun mats are displayed in Figure 6. Typical curves can be found in Supplementary Material.

Figure 6 depicts the decrease in the advancing contact angles (ACA) as the ratio of sisal/PET in the composition of these materials increased. Therefore, the addition of sisal fiber led to formation of hydrophilic materials with surface properties considerably different from those shown by PET_ref_.

The ACA ranged from 134.6 ± 2.4° (PET_ref_), which indicated a highly hydrophobic surface, to 32.5 ± 2.3° (highly hydrophilic surface, Sisal/PET_0.40_). The significant decrease in ACA values and the small values of receding contact angle (RCA) compared to those presented by PET_ref_ (Figure 6) indicated that in the Sisal/PET-mats, many polar groups—mostly those from the cellulose structure (major sisal fiber component)—were oriented towards the surface.

The increase in the average porosity of the sisal-based mats compared to that of PET_ref_ (Figure 5c) may have also contributed to the increase in the hydrophilicity of these materials. Therefore, the addition of different ratios of sisal fiber/recycled PET in the composition of the electrospun mats allows control and modulation of the surface properties of the materials for specific applications.

### 3.3. Dynamic Mechanical Analysis (DMA)

**Figure 7:**
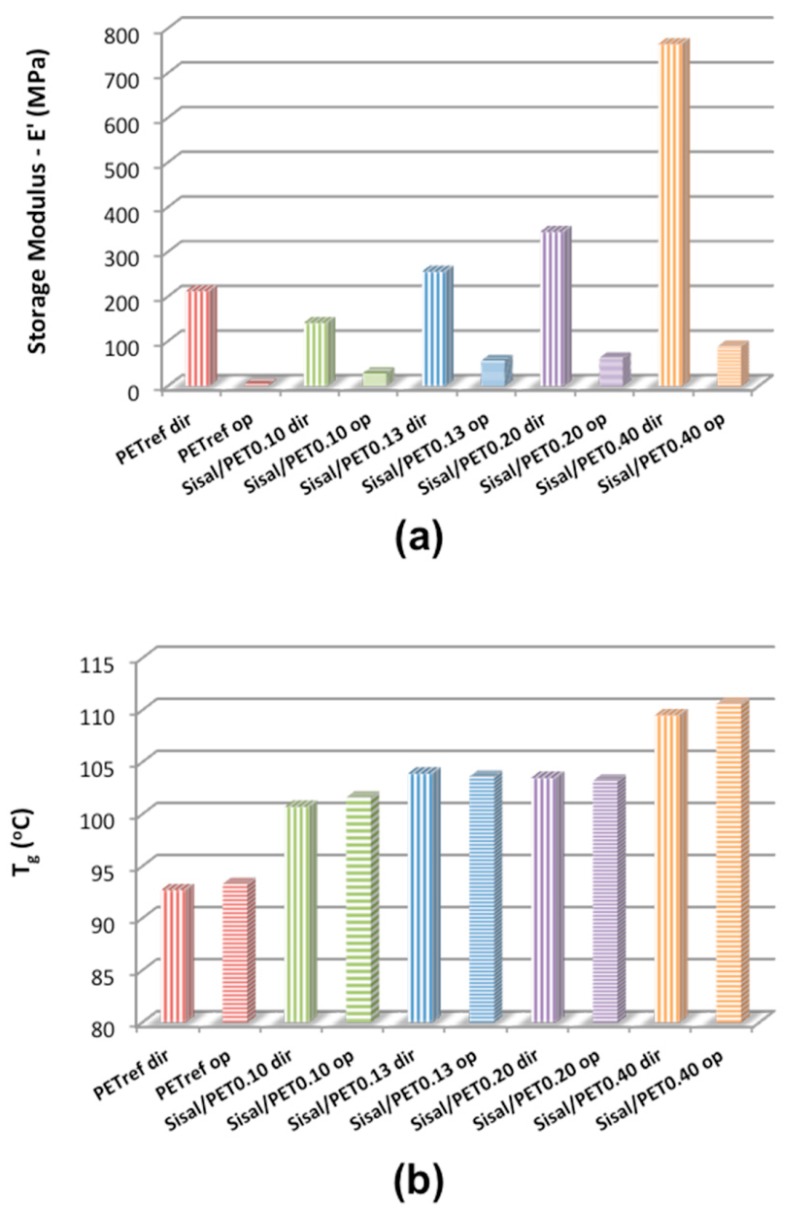
(**a**) Storage Modulus (at 30 °C) (errors from ±0.90 (Sisal/PET_0.13_*op*) to ±50.10 (Sisal/PET_0.13_*dir*)) and (**b**) *T*_g_ values (errors from ±0.04 (PET_ref_*op*) to ±0.90 (Sisal/PET_0.20_*op*)) of the electrospun mats. All materials were characterized in the preferential direction of fiber alignment (acronyms containing “*dir*”), and in the opposite direction of fiber alignment (acronyms containing “*op*”).

Figure 7a presents the storage modulus values (at 30 °C) of the electrospun mats. The results of the glass transition temperature for the recycled PET (*T*_g_ obtained from the maximum of the tan δ curve) are displayed in Figure 7b. As mentioned, the mats were evaluated with the samples positioned both in the direction (*dir*) of and in opposition (*op*) to the alignment of the ultrathin and nanofibers. Typical curves can be found in Supplementary Material.

An increase in the *E*’ values (at 30 °C) with the increase in the sisal/PET ratio in the composition of the electrospun mats can be observed from Figure 7a. In the materials characterized in the preferential direction of fiber alignment, PET_ref_*dir* presented a lower value of *E*’ (212 ± 10.2 MPa) than Sisal/PET_0.40_*dir* (*E*’ = 765 MPa), indicating that the sisal fiber (mainly the cellulose component) was responsible for the increase in *E*’. The storage modulus is related to the stiffness during load. These results indicated that the presence of sisal components in the composition of the electrospun mats led to strong interactions between the recycled polymer and sisal components at the molecular level, increasing the stiffness of the mats [43].

The values of *E*’ (at 30 °C) in Figure 7a for the materials characterized in the preferred direction of fiber alignment were higher than those presented by the materials of corresponding compositions characterized in the opposite direction. These results indicated that the alignment of the fibers relative to the force applied positively impacted the resistance of the material against the applied load, as well as the capacity of the material to recover its shape.

Thus, it can be concluded that there was a significant effect of the fiber orientation on the property of *E*’ and hence on the stiffness of the material.

It can be observed in Figure 7b that the incorporation of sisal fibers into the mat composition led to an increase in the PET *T*_g_ values; e.g., the PET_ref_*dir* presented a *T*_g_ of 92.7 ± 0.3 °C and Sisal/PET_0.40_*dir* presented a *T*_g_ value of 109.5 ± 0.8 °C. The increase in the sisal fiber/PET ratio in the mat composition favored the establishment of strong intermolecular interactions—i.e., hydrogen bond interactions and hydrophobic interactions—between these components, which progressively hampered the rotational movements of the covalent bonds present in the segments of the polymer chain [44]. The variation observed in the *T*_g_ values of the mats reinforced was similar to that observed in the storage modulus results in Figure 7a, which indicated that the interactions between the segments of the PET chain and the sisal components occurred at the molecular level. These results indicated that hybrid fibers (in which intermolecular distances favored strong interactions between the polar and apolar groups of PET segments and sisal components) were produced via electrospinning and that the fraction of hybrid fibers produced impacted the *T*_g_ of PET.

According to Figure 7b, there was no significant difference in *T*_g_ values between the mats with the same composition but characterized in the preferential direction of fiber alignment and in the opposite direction. The glass transition is a consequence of the movement of segments of the PET chains at the molecular level, and the alignment of the fibers in the direction of or in opposition to the applied load did not influence the movement of the segments.

### 3.4. Tensile Tests

**Figure 8:**
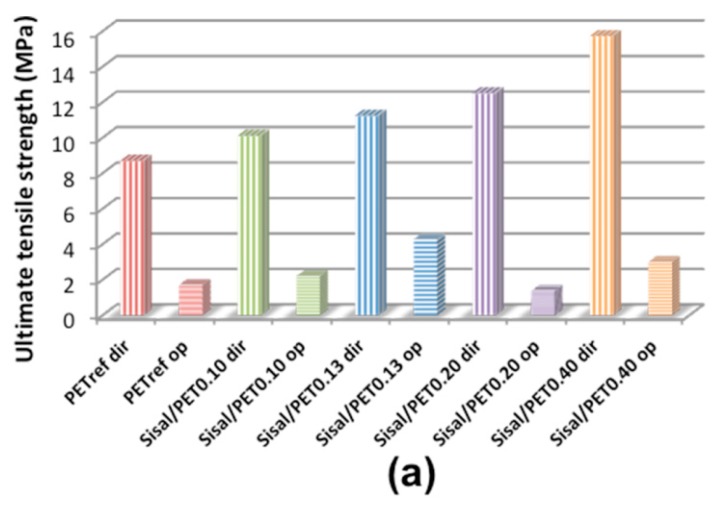

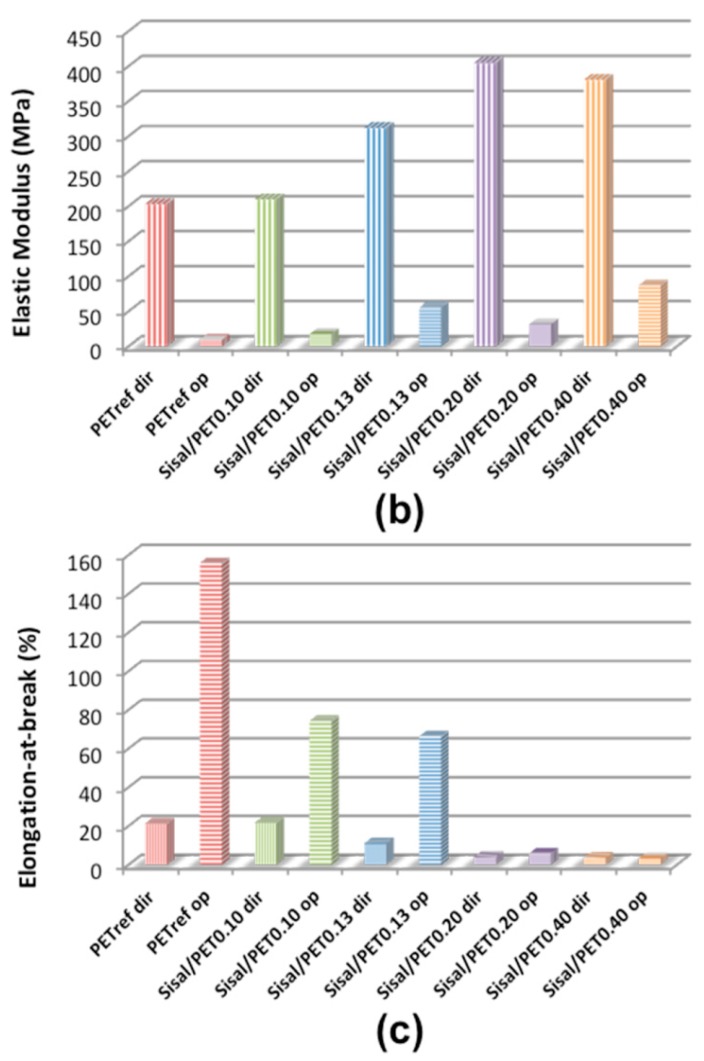
(**a**) Ultimate tensile strength (errors from ±0.10 (Sisal/PET_0.10_*op*) to ±1.20 (Sisal/PET_0.10_*dir*)); (**b**) elastic modulus (errors from ±0.10 (Sisal/PET_0.13_*op*) to ±15.63 (Sisal/PET_0.40_*dir*)); and (**c**) elongation at break (errors from ±0.01 (Sisal/PET_0.20_*dir*) to ±17.53 (Sisal/PET_0.13_*op*)) of the electrospun mats. All materials were characterized in the preferential direction of fiber alignment (acronyms containing “*dir*”), and in the opposite direction of fiber alignment (acronyms containing “*op*”).

Figure 8 presents the results of tensile tests regarding the rupture strength, elastic modulus, and elongation at break of the electrospun mats. All materials were characterized in the perpendicular direction relative to the collector axis (preferential direction of fiber alignment) and in the parallel direction (opposite direction of fiber alignment). Typical curves can be found in Supplementary Material.

It can be observed in Figure 8a that an increase in the tensile strength values occurred with the increase in the sisal/PET ratio in the composition of the electrospun mats, especially for those characterized in the preferential direction of fiber alignment. Regarding these mats, PET_ref_*dir* presented the lowest value of tensile strength (8.7 ± 0.5 MPa), and Sisal/PET_0.40_*dir*, with the highest ratio of sisal/PET in its composition, presented the highest value for this property (15.72 ± 0.2 MPa). These results indicated that sisal fiber (mainly the cellulose component) was responsible for the increase in the tensile strength values [45]. Thus, it can be concluded that the presence of cellulose, hemicelluloses, and lignin in the composition of the materials favored the establishment of strong intermolecular interactions between this polysaccharide and PET chains, making the orientation of the polymer chains with the traction axis during the final stage of deformation difficult, causing an increase in mechanical resistance of the electrospun mats.

The tensile strength values of the materials characterized in the preferential direction of fiber alignment (Figure 8a) were superior and more divergent compared to the values of this property presented by the mats of the corresponding composition characterized in the opposite direction. These results are probably a consequence of the fact that when a mechanical force is applied in the preferential direction of fiber alignment, the fibers only need a minimum rotation to align with the direction of the applied force. Consequently, a high percentage of fibers readily resists the force applied, endowing the material with a high tensile strength. Conversely, when the force is applied in the direction opposite of fiber alignment, a greater rotation and reorientation of the fibers are necessary to align with the axis of the applied force, which leads to low tensile strength.

In general, the elastic modulus of the electrospun mats (Figure 8b) exhibited the same trend as that observed for *E*’ (Figure 6b) and tensile strength (Figure 8a), evidencing the influence of the increasing sisal/PET ratio on the composition of the materials and the influence of fiber orientation on the mechanical properties. According to Figure 8b, PET_ref_*op* presented the lowest value of elastic modulus (9.5 ± 2.5 MPa), and Sisal/PET_0.40_*dir*, with the highest ratio of sisal/PET in its composition, presented one of the highest values for this property (380.55 ± 15.63 MPa).

An opposite trend to that presented for *E*’ (at 30 °C) (Figure 7a), tensile strength (Figure 8a) and elastic modulus (Figure 8b) were observed regarding the values of elongation at break for the electrospun mats (Figure 8c). The mats with lower sisal/PET ratios in their compositions characterized in the direction opposite to fiber alignment presented the highest values of elongation at break (Figure 8c). When a mechanical force is applied in the direction opposite to fiber alignment, a greater rotation and reorientation of the fibers are required to align them with the axis of the applied tension, as mentioned above, which leads to higher elongation of these materials compared to that in situations in which the mechanical force is applied in the fiber alignment direction [46,47]. According to Figure 7c, PET_ref_*op* presented the highest value of elongation at break (156 ± 2%), and Sisal/PET_0.40_*dir*, with the highest ratio of sisal/PET in its composition, presented one of the lowest values for this property (3.6 ± 0.0%).

## 4. Conclusions

Ultrathin and nanoscale aligned fibers were successfully prepared via electrospinning of solutions based on lignocellulosic sisal fibers and recycled PET with different ratios of sisal/PET in their compositions. The SEM images, as well as the average diameters and average porosities, indicated that mats composed of networks consisting of PET fibers, of the components of the lignocellulosic fibers, and of hybrid fibers (PET/cellulose-hemicelluloses-lignin) were obtained. These results, together with those from contact angle measurements, showed that it was possible to control the fiber diameters and porosities and the hydrophilic/hydrophobic character of the mats using different ratios of sisal fiber/PET to prepare solutions subsequently submitted to electrospinning. The possibility of adjusting these parameters is of importance for several applications, for example, for the use of mats as enzyme supports.

The results of DMA and tensile tests revealed that fiber alignment in the direction of the applied force had a strong influence of and that the increase in the sisal fiber/PET ratio positively impacted the storage and tensile moduli, as well as the tensile strength of the mats. These results are of particular importance for applications in which mechanical properties are important, such as in filter systems and as reinforcements in nanocomposites.

This set of results discloses the potential of obtaining mats with diversified properties, opening an opportunity for a wide range of applications. The approach of this study, to our knowledge, is unprecedented.

## Figures and Tables

**Table 1:** Composition and respective reference codes of the electrospun mats.

Sisal fiber (g)	Recycled PET (g)	Sisal/PET ratio	Sample code
-	0.45	Reference sample	PET_ref_
0.06	0.60	0.10	Sisal/PET_0.10_
0.06	0.45	0.13	Sisal/PET_0.13_
0.06	0.30	0.20	Sisal/PET_0.20_
0.06	0.15	0.40	Sisal/PET_0.40_

## Supplementary Materials

The supplementary materials are available online at http://www.mdpi.com/2073-4360/10/5/538/s1.
