# Supplementary material for: Renewable Resources and a Recycled Polymer as Raw Materials: Mats from Electrospinning of Lignocellulosic Biomass and PET Solutions
Source: Polymers (Basel). 2018 May 17;10(5):538. doi: 10.3390/polym10050538 (PMC6415374; doi:10.3390/polym10050538)
Supplement: Supplementary file 1 [file polymers-10-00538-s001.pdf]

Supplementary Material:

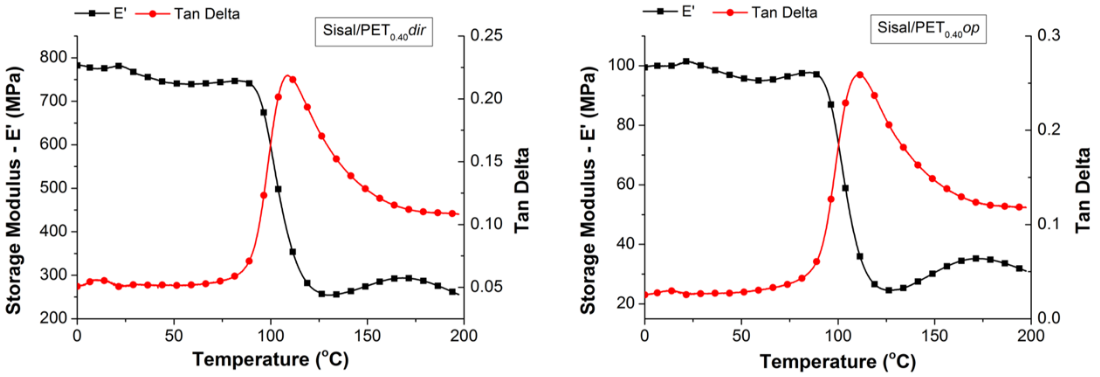

Figure S1: DMA curves of Sisal/$PET_{0.40}$ (*dir and op*)

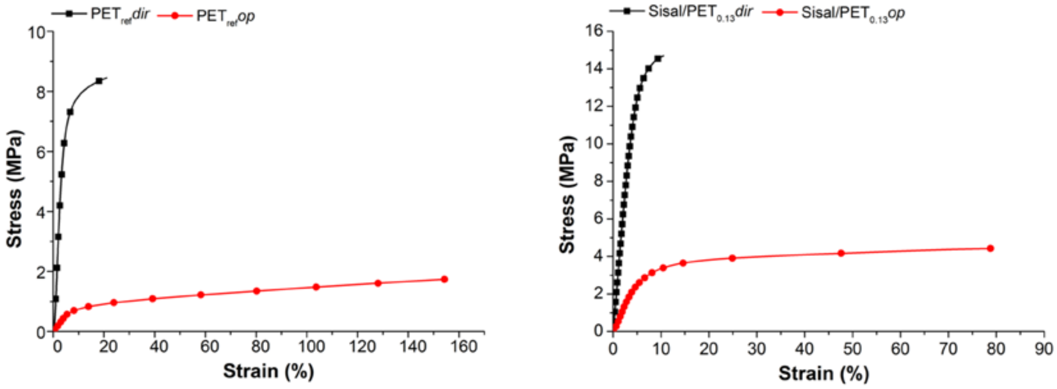

Figure S2: Tensile strength curves of PET_{ref} and Sisal/$PET_{0.40}$ (*dir and op*)

.

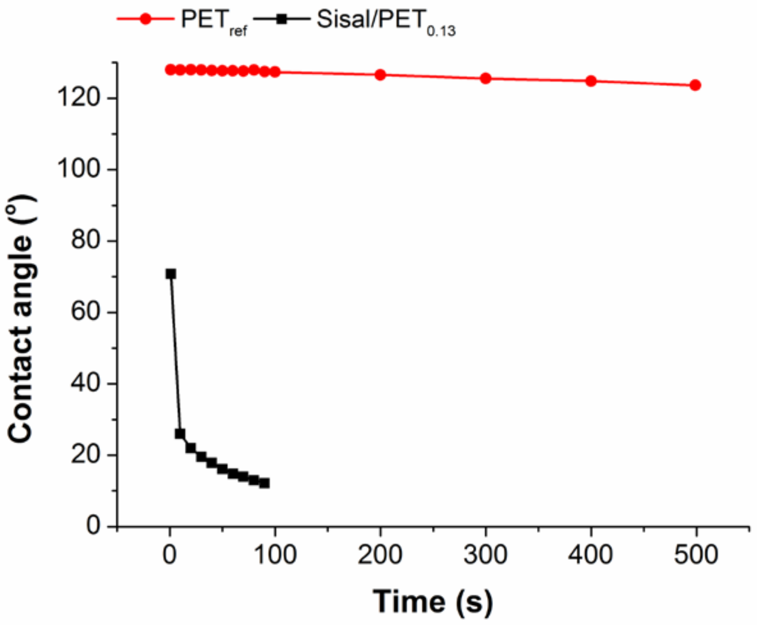

Figure S3: Contact angle curves of PET_{ref} and Sisal/$PET_{0.13}$.
